# Antioxidant and Antiproliferative Activity of Finasteride against Glioblastoma Cells

**DOI:** 10.3390/pharmaceutics13091410

**Published:** 2021-09-06

**Authors:** Hyeon Ji Kim, Tae-Jun Kim, Yu Gyung Kim, Chaeeun Seong, Jin-Hwa Cho, Wanil Kim, Kyung-Ha Lee, Do-Yeon Kim

**Affiliations:** 1 Department of Pharmacology, School of Dentistry, Kyungpook National University, Daegu 41940, Korea; guswl1634@naver.com (H.J.K.); toy5988@naver.com (T.-J.K.); cosmos0468@naver.com (Y.G.K.); qjdo@naver.com (C.S.); cjinhwa@knu.ac.kr (J.-H.C.); 2 Department of Biochemistry, Department of Convergence Medical Science, Institute of Health Sciences, College of Medicine, Gyeongsang National University, Jinju 52727, Korea; 3 Division of Cosmetic Science and Technology, Daegu Haany University, Gyeongsan 38610, Korea

**Keywords:** glioblastoma, finasteride, proliferation, β-catenin

## Abstract

Glioblastoma is an actively growing and aggressive brain tumor with a high propensity of recurrence. Although the surgical removal of tumor mass is the primary therapeutic option against glioblastoma, supportive pharmacotherapy is highly essential due to incredibly infiltrative characteristic of glioblastoma. Temozolomide, an FDA-approved alkylating agent, has been used as a first-line standard pharmacological approach, but several evident limitations were repeatedly reported. Despite additional therapeutic options suggested, there are no medications that successfully prevent a recurrence of glioblastoma and increase the five-year survival rate. In this study, we tested the possibility that finasteride has the potential to be developed as an anti-glioblastoma drug. Finasteride, an FDA-approved medication for the treatment of benign prostate hyperplasia and androgenic alopecia, is already known to pass through the blood–brain barrier and possess antiproliferative activity of prostate epithelial cells. We showed that finasteride inhibited the maintenance of glioma stem-like cells and repressed the proliferation of glioblastoma. Mechanistically, finasteride lowered intracellular ROS level by upregulating antioxidant genes, which contributed to inefficient β-catenin accumulation. Downregulated β-catenin resulted in the reduction in stemness and cell growth in glioblastoma.

## 1. Introduction

Glioblastoma is highly aggressive and devastating primary brain tumor. Although it has been regarded as a rare cancer with comparatively low incidence (approximately 5 per 100,000 persons) [1], glioblastoma is infamous for short median survival time (12 to 18 months) and desperate five-year survival rate (6.8%) [2]. The standard of care for glioblastoma is maximal surgical resection, followed by concurrent radiation and chemotherapy [3]. Notably, due to extremely infiltrative nature of glioblastoma, residual infiltrating tumor cells commonly exist surrounding the visible tumor mass, which are not identified by radiographic imaging [4]. For this reason, pharmacotherapy is strongly required after surgical management for glioblastoma patients.

Temozolomide is an FDA-approved alkylating agent, which triggers apoptosis of glioblastoma cells. Compared to radiotherapy alone, radiation plus concomitant and adjuvant temozolomide provided 2.5 months of the median survival benefit and significantly improved the two-year survival rate from 10.4% to 26.5% [5], leading to the use of temozolomide as a first-line standard approach against glioblastoma. However, several evident limitations have been identified on temozolomide so far. Due to its pharmacokinetically unstable property in plasma, systemic long-term administration of temozolomide with a high dosage is demanded, which causes high levels of toxicities including thrombocytopenia, leukopenia, and neutropenia [6]. The acquisition of temozolomide resistance is another important obstacle for glioblastoma treatment. For example, tumor cells often abundantly express O6-methylguanine-DNA methyl transferase (MGMT), a protein that is responsible for restoration of DNA damage, to suppress apoptotic pathway [7]. In addition, glioblastoma cells occasionally utilize the autophagy system to develop drug resistance [8]. Above all things, temozolomide ultimately failed to prevent recurrence of glioblastoma and increase the five-year survival rate.

Owing to these drawbacks of temozolomide, additional therapeutic approaches have been developed. The use of carmustine, an alkylating drug that belongs to the nitrosourea family, has provided 2–4 months of the overall survival benefit [9]. Although carmustine was FDA-approved for the treatment of newly diagnosed high-grade glioma and recurrent glioblastoma, the safety issue is still under debate. Lomustine is another alkylating nitrosourea derivative that has recently been defined as a standard of care for glioblastoma in Europe [10]. However, lomustine is shown to be effective only in recurrent glioblastoma, and anti-tumor activity is restricted to patients with cancers harboring methylated MGMT promoter [11]. Targeted therapy was also devised for the treatment of glioblastoma. Bevacizumab is a monoclonal antibody targeting VEGF and already approved by the FDA against recurrent glioblastoma in 2009 [12]. However, accumulating evidence has shown that bevacizumab failed to highly improve the overall survival of glioblastoma patients [13]. Therefore, there have still been constant demands for the development of new therapeutic options against glioblastoma.

Drug repurposing (or repositioning) has been the center of attention because it would be a fast track to develop new medications in a cost- and time-effective way. Particularly, however, finding efficacious drugs for central nervous system disorders, including glioblastoma, would be extremely difficult and risky due to the existence of the blood–brain barrier as an additional hurdle. As FDA-approved medication for the treatment of benign prostate hyperplasia and androgenic alopecia, finasteride was already proven to penetrate the blood–brain barrier. Furthermore, given that finasteride has been clinically used to treat benign prostate enlargement, it would possess a suppressive activity against cell proliferation. Indeed, there have been a few attempts to test finasteride as a new glioblastoma drug, but the detailed intracellular mechanisms of finasteride in glioblastoma cells have not been determined yet. In this study, we tested a possibility that finasteride has the potential to be developed as an anti-glioblastoma drug.

## 2. Materials and Methods

### 2.1. Cell Culture

U373 and T98G human glioblastoma cells were purchased from Korea Cell Line Bank (KCLB, Seoul, Korea) and maintained in Minimum Essential Medium (Gibco) supplemented with 10% fetal bovine serum (FBS) and 1% penicillin-streptomycin, according to the KCLB cell culture guidelines (https://cellbank.snu.ac.kr, accessed on 1 March 2017, 9 April 2020). To enrich glioblastoma stem-like cell populations, U373 and T98G cells were adapted in N2 culture medium supplemented with 1% FBS for 6 days and then transferred to the serum-free N2 culture media supplemented with 20 ng/mL EGF and bFGF.

### 2.2. Drug

Temozolomide (Sigma Aldrich, St. Louis, MO, USA, cat no. T2577) and finasteride (Sigma Aldrich, St. Louis, MO, USA, cat no. F1293) were purchased from Sigma-Aldrich and dissolved in DMSO. Temozolomide was treated at a final concentration of 150 μM (U373) or 400 μM (T98G), unless otherwise stated.

### 2.3. Transfection and Reporter Assays

For plasmid transfection, cells were dissociated and plated in culture media. After a 24 h incubation, EGFR promoter reporter plasmid (Genecopoeia, Rockville, MD, USA, cat no. HPRM45993-PG02) or M50 Super 8x TOPFlash reporter plasmid (a gift from Randall Moon, Addgene, Watertown, MA, USA, plasmid # 12456) was co-transfected with a TK-Renilla plasmid using Lipofectamine 3000 (Life Technologies, Carlsbad, CA, USA) according to the manufacturer’s instruction. At 12 h post-transfection, cells were treated with DMSO or finasteride for additional 24 h. Harvested cells and culture media were used to analyze the activities of firefly luciferase and *Gaussia* luciferase, respectively. Relative luciferase activity was shown as the ratio of firefly or *Gaussia* to Renilla activity. For data presentation, relative luciferase activities of DMSO-treated cells were set to 1.

### 2.4. Protein Preparation and Immunoblot Analysis

Cells were directly disrupted in laemmli buffer (60mM Tris-HCl (pH 6.8), 2% (*w/v*) sodium dodecyl sulfate (SDS), 10% (*v/v*) glycerol, 0.02% (*w/v*) bromophenol blue), followed by sonication and heat-denaturation. Samples were separated on SDS polyacrylamide gels, and then, proteins were transferred to a poly-vinylidene fluoride (PVDF) membrane. Immunoblot assays were performed with antibodies against GAPDH (Cusabio, Houston, TX, USA, cat no. CSB-MA000184), β-catenin (Thermo Fisher Scientific, Waltham, MA, USA, cat no. MA1-300), phospho H3 at Ser 10 (Cell Signaling, Leiden, The Netherlands, cat no. 3377), phospho AKT (Thermo Fisher Scientific, Waltham, MA, USA, cat no. 700392), total AKT (Cell Signaling, Leiden, The Netherlands, cat no. 9272), SESN2 (Abcam, Cambridge, UK, cat no. ab178518), SOD2 (Cusabio, Houston, TX, USA, cat no. CSB-PA022398LA01HU), PRDX5 (Bethyl Laboratories, Montgomery, TX, USA, cat no. A305-339A), γH2AX (Novus Biologicals, Centennial, CO, USA, cat no. NB100-384), EGFR (Bioworld Technology, Bloomington, MN, USA, cat no. BS1533), P62 (Abcam, Cambridge, UK, cat no. ab56416), phospho P70S6K (Cell Signaling, Leiden, The Netherlands, cat no. 9205), total P70S6K (Cell Signaling, Leiden, The Netherlands, cat no. 9202), phospho 4EBP1 (Cell Signaling, Leiden, The Netherlands, cat no. 2855), and total 4EBP1 (Cell Signaling, Leiden, The Netherlands, cat no. 9644). Immunoreactive signals were detected with the D-Plus^TM^ ECL Femto system (Dongin LS, Hwaseong, Korea).

### 2.5. Immunofluorescence

Cells were fixed with 4% paraformaldehyde and permeabilized with 0.2% Triton X-100 for 15 min each at RT. After blocking samples with 2% BSA for 30 min at RT, cells were subjected to immunostaining with antibodies against phospho H3 at Ser 10 (Cell Signaling, Leiden, The Netherlands, cat no. 3377) and γH2AX (Novus Biologicals, Centennial, CO, USA, cat no. NB100-384). The next day, cells were incubated with Flamma^®^552-conjugated goat anti-rabbit IgG (bioacts, Incheon, Korea) for 30 min at RT. The fluorescence signals were visualized with EVOS FL Auto Imaging System (Thermo Fisher Scientific, Waltham, MA, USA).

For bromodeoxyuridine (BrdU) staining, cells were labeled with BrdU by incubation with 10 μM of BrdU for 30 min. After fixation and permeabilization as described above, cells were treated with 2M HCl for 20 min and neutralized with 0.1M sodium borate buffer for 30 min. After blocking, cells were subjected to immunofluorescence staining with anti-BrdU (Thermo Fisher Scientific, Waltham, MA, USA, cat no. MA3-071) primary antibody. The next day, cells were incubated with Flamma^®^488-conjugated goat anti-mouse IgG (bioacts, Incheon, Korea) for 30 min at RT. Signal visualization was performed as described above.

### 2.6. ROS Detection

Intracellular ROS levels were determined by using the fluorogenic CellROX^®^ Orange reagent (Invitrogen, Waltham, MA, USA, cat no. C10443) according to the manufacturer’s instruction. Cells were plated in a 12-well plate and treated with DMSO or finasteride. At 24 h post-treatment, CellROX^®^ reagent was added to a final concentration of 5 μM. Microscopy images were taken using EVOS FL Auto Imaging System (Thermo Fisher Scientific, Waltham, MA, USA).

### 2.7. Monitoring of the Mitochondrial Membrane Potential

The mitochondrial membrane potential was analyzed by using TMRE fluorescence (Abcam, Cambridge, UK, cat no. ab113852). Cells were loaded with 100 nM TMRE at 24 h after DMSO or finasteride treatment to assess the effects of finasteride on ΔΨm of glioblastoma cells. The fluorescence signals were visualized with EVOS FL Auto Imaging System (Thermo Fisher Scientific, Waltham, MA, USA).

### 2.8. Quantitative Real-Time RT-PCR

Total RNA was extracted from cultured cells using an RNA Purification Kit (Favorgen, Ping-Tung, Taiwan), and 200 ng of total RNA was treated with RNase-free DNase (Sigma Aldrich, St. Louis, MO, USA) for 15 min. After inactivation of DNase with EDTA treatment and heating, total RNA was reverse transcribed into cDNA using First Strand cDNA Synthesis Kit (Thermo Fisher Scientific, Waltham, MA, USA). Quantitative RT-PCR was performed on cDNA samples with Luna^®^ universal qPCR master mix (New England Biolabs, Ipswich, MA, USA) on the Mic qPCR Cycler (bio molecular systems, Queensland, Australia). The relative mRNA level was quantitated as values of 2^(Ct[RPL32] − Ct[gene of interest])^. The sequences of the forward and reverse primers are listed in Table 1.

### 2.9. Statistical Analysis

The unpaired two-tailed Student’s *t*-test was used for experiments comparing two sets of data unless noted. All results are expressed as mean±s.e.m. GraphPad Prism software (version 6, San Diego, CA, USA) was used for all statistical analysis. Differences were considered significant when * *p* < 0.05, ** *p* < 0.01, and *** *p* < 0.001.

## 3. Results

### 3.1. High-Dose Finasteride Downregulates β-Catenin Protein Level

**Figure 1:**
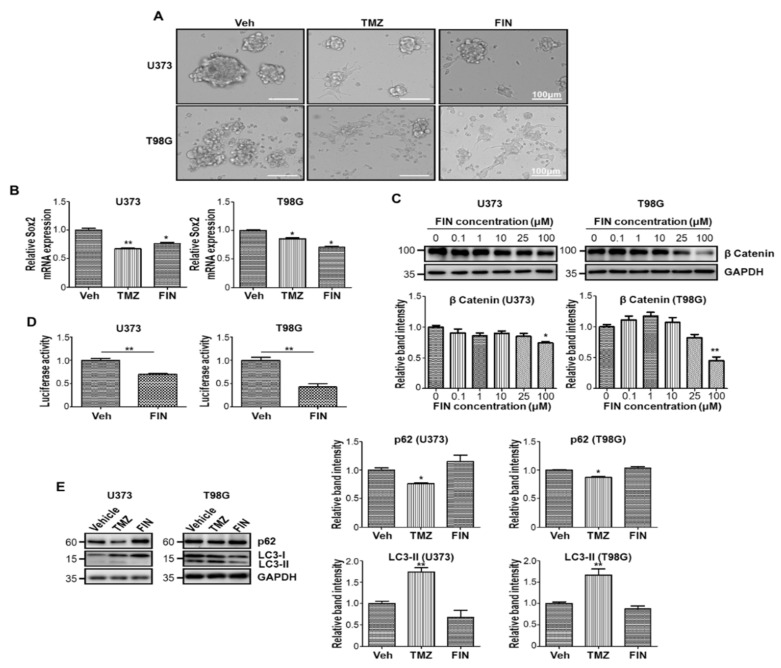
Glioblastoma stem-like cells are sensitive to high-dose finasteride. (**A**) Cell morphology of U373 (upper) and T98G (lower) glioblastoma cells under sphere-forming culture condition upon treatment of DMSO or temozolomide (TMZ) or 100 μM finasteride (FIN) for 24 h. (**B**) The mRNA expression of Sox2 in U373 (left) and T98G (right) cells upon vehicle or drug treatment, as measured by quantitative RT-PCR analysis. The mRNA level of vehicle-treated cells was set to 1. (**C**) Immunoblot analysis of β-catenin in U373 (left) or T98G (right) cells after treatment with vehicle or FIN at the indicated concentration for 24 h. GAPDH was used as a loading control. The relative band intensities of β-catenin are shown below. The intensities of vehicle-treated samples were arbitrarily set to 1. (**D**) β-catenin activity was measured in U373 (left) or T98G (right) cells by TOPFlash reporter assay upon treatment of DMSO or 100 μM FIN for 24 h. (**E**) Immunoblot analysis of P62 and LC3 in U373 (left) or T98G (right) cells after vehicle or drug treatment for 24 h. The relative band intensities of P62 and LC3-II are represented. The intensities of vehicle-treated samples were arbitrarily set to 1. * *p* < 0.05, ** *p* < 0.01.

To determine the anticancer activity of finasteride, we tested the effect of finasteride on glioblastoma stem-like cells, which has not been demonstrated so far. Previously, we showed that cancer stem-like cell populations were enriched by maintaining cells with serum-free culture media supplemented with epidermal growth factor (EGF) and basic fibroblast growth factor (bFGF) [14]. Under this sphere-forming condition, we treated two different glioblastoma cells, U373 and T98G, with finasteride and temozolomide. As earlier reported, temozolomide suppressed the sphere-forming capacity of both glioblastoma cells [15]. Interestingly, similar to temozolomide, finasteride reduced the sphere size of glioblastoma or even made cells grow in monolayer, suggesting that finasteride suppresses the stemness of cancer stem-like cells in glioblastoma (Figure 1A). The inhibitory function of finasteride in the maintenance of the stemness was confirmed by qRT-PCR, showing that the mRNA level of Sox2, a marker for stem-like tumor cells, was downregulated by finasteride treatment (Figure 1B).

Given that (1) β-catenin has been regarded as critical factor in diverse cancer cells/cancer stem cells [16] and (2) Sox2 governs the stem cell fate downstream of Wnt/β-catenin signaling [17], we examined the level of β-catenin under finasteride treatment. As a result, finasteride clearly lowered β-catenin protein abundance at a high concentration in both U373 and T98G cells (Figure 1C). Consistently, when we measured TCF/Lef/β-catenin-dependent transcriptional activity by utilizing the TOFlash reporter system [18], β-catenin-responsive luciferase expression was significantly repressed by finasteride in both glioblastoma cells (Figure 1D). Accumulating evidence has shown that temozolomide induced autophagy, and Wnt/β-catenin signaling works as a key downstream player [19]. In line with these reports, p62 level was declined by temozolomide treatment, mirroring an activation of autophagy flux. In contrast, finasteride did not alter p62 abundance, suggesting that it does not prompt autophagy process, possibly due to β-catenin downregulation (Figure 1E). Taken together, high-dose finasteride dampens the β-catenin protein level, leading to a reduction in glioblastoma stemness.

### 3.2. High-Dose Finasteride Shows Antiproliferative Function against Glioblastoma

To precisely determine the molecular and cellular function of finasteride, we performed the gene ontology (GO) enrichment analysis and biological pathway analysis with publicly available transcriptome datasets [20,21,22]. Previously, Fielden et al. analyzed in vivo transcriptome after administration of 147 non-genotoxic compounds, including finasteride, in rats [23]. By further examination with this expression profile data, 851 genes (642 upregulated, 209 downregulated) were turned out to be differentially expressed genes (DEGs) upon daily finasteride treatment for 5 days. Notably, the GO analysis showed that downregulated genes were highly enriched in positive cellular process and homeostasis such as proliferation (Supplementary Figure S1A), suggesting that finasteride was likely to suppress cell-proliferating potential. Meanwhile, diverse cellular processes including ion transport, localization, secretion, and G protein-coupled receptor-related signaling surfaced when the analysis was performed with upregulated genes. Interestingly, the biological pathway analysis indicated that the downregulated gene set was significantly enriched in signaling pathways in glioblastoma, suggesting an antitumor activity of finasteride (Supplementary Figure S1B).

**Figure 2:**
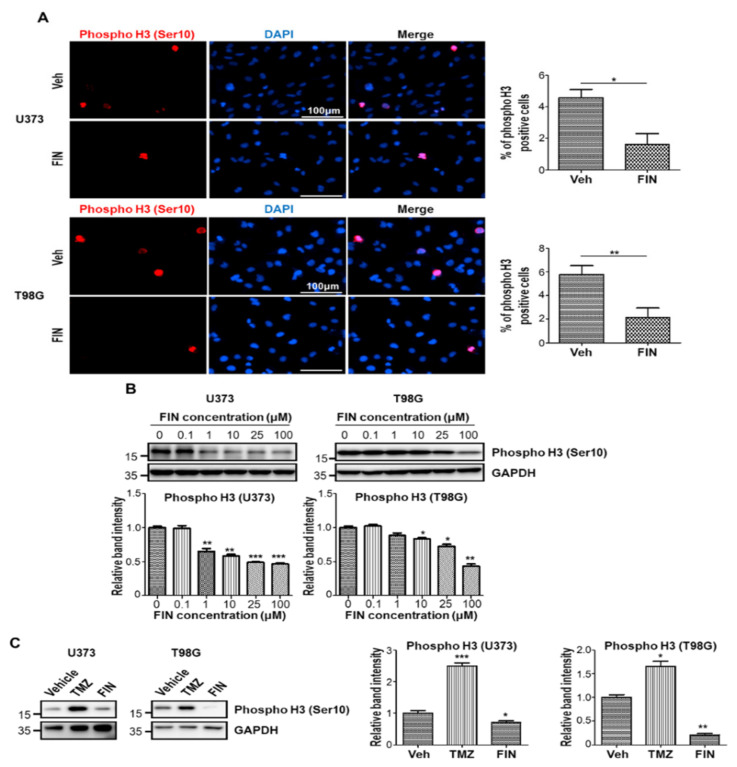
High-dose finasteride suppresses the proliferating capacity of glioblastoma cells. (**A**) U373 (upper) and T98G (lower) cells were treated with vehicle or 100 μM finasteride (FIN) for 24 h, and they were immunostained with a phospho histone 3 (Ser 10)-specific antibody (red). Nuclear DAPI (4′, 6-diamidino-2-phenylindole) signal is presented in blue (left). The quantification of phospho histone 3-positive cell proportions in vehicle- or FIN-treated cells (right). (**B**) Immunoblot analysis of phospho histone 3 in U373 (left) or T98G (right) cells after treatment with vehicle or FIN at the indicated concentration for 24 h. GAPDH was used as a loading control. The relative band intensities of phospho histone 3 are shown below. The intensities of vehicle-treated samples were arbitrarily set to 1. (**C**) Immunoblot analysis of phospho histone 3 in U373 (left) or T98G (right) cells upon treatment of DMSO or temozolomide (TMZ) or 100 μM FIN for 24 h. GAPDH was used as a loading control. The relative band intensities of phospho histone 3 are represented. The intensities of vehicle-treated samples were arbitrarily set to 1. * *p* < 0.05, ** *p* < 0.01, *** *p* < 0.001.

Based on the GO analysis result and previous reports demonstrating the role of β-catenin in cell cycle progression [24], we evaluated the antiproliferative capacity of finasteride on glioblastoma by performing immunofluorescence on U373 and T98G cells, using an antibody against the phospho Histone 3 (Ser10) after treatment with either vehicle or finasteride for 24 h. As a result of counting random fields of view, we showed that the percentage of phospho Histone 3-positive cells was dramatically decreased by finasteride treatment (Figure 2A). Consistently, high-dose finasteride efficiently dampened the level of phospho Histone 3 protein (Figure 2B). Clearly, compared to temozolomide that did not reduce phospho Histone 3 level, finasteride dramatically lowered phospho Histone 3 level, meaning that finasteride showed stronger antiproliferative potential over temozolomide, the Food and Drug Administration (FDA)-approved medication for glioblastoma multiforme patients (Figure 2C). To further confirm the antiproliferative activity of finasteride on glioblastoma cells, we additionally performed BrdU incorporation assay in vehicle or finasteride-treated cells. Immunofluorescent staining for BrdU showed that the percentage of BrdU-positive cells significantly decreased following finasteride treatment in both U373 and T98G cells (Supplementary Figure S2). Taken together, finasteride showed the antiproliferative capacity in glioblastoma cells.

### 3.3. Finasteride Diminishes Intracellular Reactive Oxygen Species (ROS) Level through Inducing the Expression of Antioxidant Genes

**Figure 3:**
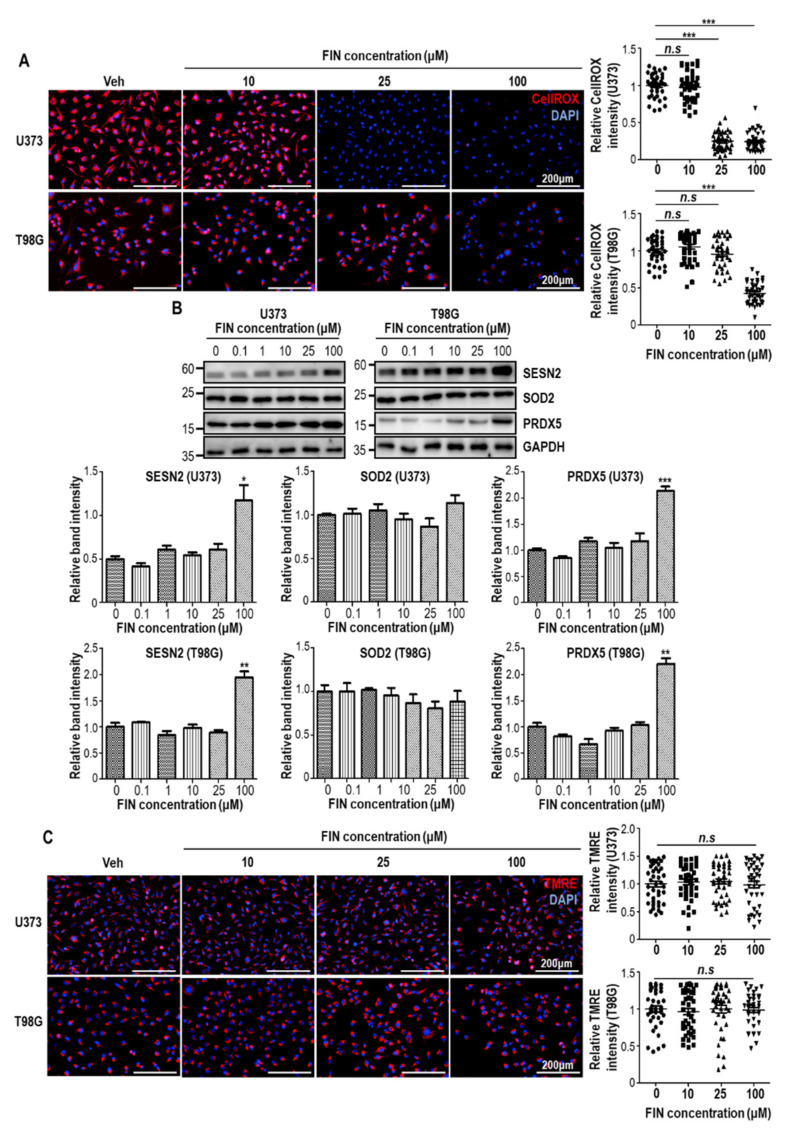
High-dose finasteride shows the antioxidant potential in glioblastoma cells. (**A**) U373 (upper) and T98G cells were subjected to CellROX staining after treatment with vehicle or finasteride (FIN) at the indicated concentration for 24 h. Nuclear DAPI signal is shown in blue. The relative CellROX signal intensities are shown (right). Each dot means an individual cell. (**B**) Immunoblot analysis of SESN2, SOD2, and PRDX5 in U373 (left) or T98G (right) cells after treatment with vehicle or FIN at the indicated concentration for 24 h. GAPDH was used as a loading control. The relative band intensities of SESN2, SOD2, and PRDX5 are shown below. The intensities of vehicle-treated samples were arbitrarily set to 1. (**C**) U373 (upper) and T98G cells were subjected to TMRE staining after treatment with vehicle or finasteride (FIN) at the indicated concentration for 24 h. Nuclear DAPI signal is shown in blue. The relative TMRE signal intensities are shown (right). Each dot means an individual cell. * *p* < 0.05, ** *p* < 0.01, *** *p* < 0.001.

Compared to normal cells, tumor cells often have higher intracellular ROS levels, mainly due to accelerated metabolic process. Increased ROS in tumors function as critical signaling molecules, leading to subsequent activation of cell growth and survival [25]. Furthermore, it was previously shown that β-catenin could be accumulated in redox-dependent manner, even without extracellular Wnts [26], raising a possibility that finasteride may influence intracellular redox status in glioblastoma cells. To this end, we investigated ROS level by CellROX staining at 24 h after finasteride treatment. Interestingly, finasteride eliminated intracellular ROS level in a dose-dependent manner (Figure 3A). To figure out the mechanism of finasteride-mediated ROS decrease, we examined expression levels of antioxidant genes. Evidently, protein levels of SESTRIN2 and PRDX5 were elevated at high concentration of finasteride in both U373 and T98G cells (Figure 3B). In contrast, SOD2 level was unaltered. Because the aberrant ROS production could be caused by dysregulated metabolic process, we monitored mitochondrial membrane potential with the TMRE fluorescent probe. However, TMRE labeling results showed that finasteride seemed not to alter metabolic flux, even at high dose of finasteride (Figure 3C). Taken together, high-dose finasteride protects ROS accumulation through the induction of antioxidant genes, such as SESTRIN2 and PRDX5. This antioxidant activity of finasteride might contribute to β-catenin downregulation and thereby antiproliferative activity, at least in part.

### 3.4. High-Dose Finasteride Suppresses AKT/mTOR Signaling in Glioblastoma Cells

**Figure 4:**
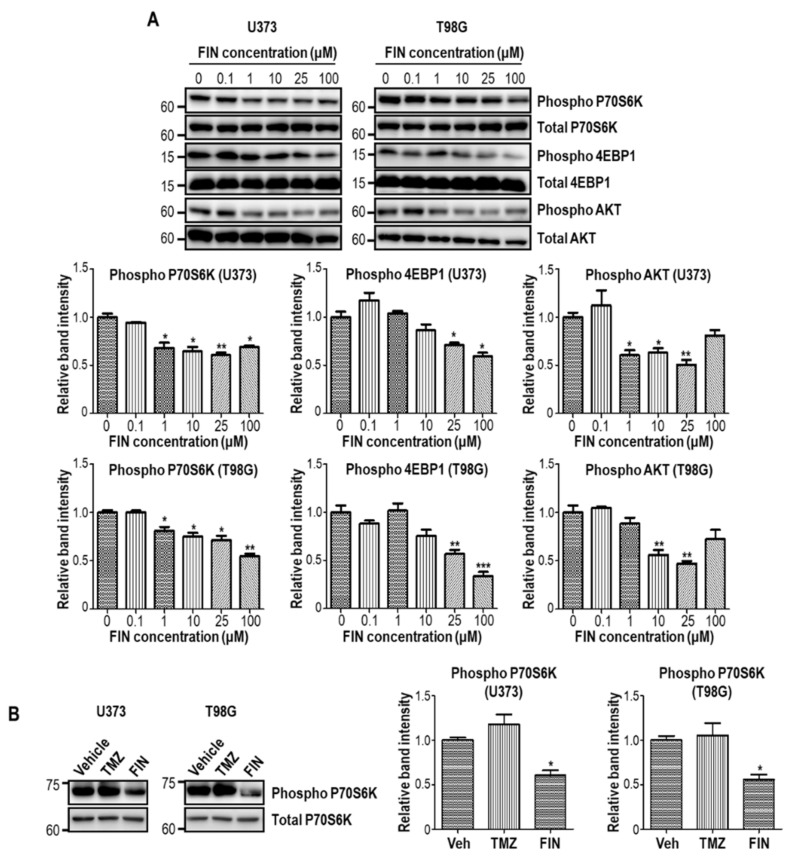
High-dose finasteride inhibits AKT/mTOR pathway in glioblastoma cells. (**A**) U373 (left) and T98G (right) cells were treated with DMSO or various concentrations of finasteride (FIN) for 24 h. Protein levels of phospho P70S6K, P70S6K, phospho 4EBP1, 4EBP1, phospho AKT, and AKT were detected by Western blot assay. The relative band intensities of the phospho proteins are shown below. The intensities of vehicle-treated samples were arbitrarily set to 1. (**B**) Immunoblot analysis of phospho P70S6K in U373 (left) or T98G (right) cells upon treatment of DMSO or temozolomide (TMZ) or 100 μM FIN for 24 h. P70S6K was used as a loading control. The relative band intensities of phospho P70S6K are represented. The intensities of vehicle-treated samples were arbitrarily set to 1. * *p* < 0.05, ** *p* < 0.01, *** *p* < 0.001.

The increased proliferative potential of tumor cells are frequently supported by AKT/mTOR signaling that contributes to the upregulated protein synthesis and cellular growth. Moreover, accumulated ROS have been reported to exaggerate AKT/mTOR signaling in some context [27]. To this end, we evaluated AKT/mTOR pathway activity upon finasteride treatment. Immunoblot assay showed that the phosphorylation of 4EBP1 and p70S6K, the representative substrates of mTOR, were significantly reduced by finasteride treatment in both U373 and T98G cells, in a dose-dependent manner (Figure 4A). In addition, AKT phosphorylation was also decreased upon finasteride administration. On the other hand, temozolomide failed to decrease the phosphorylation level of p70S6K (Figure 4B) as well as Histone 3 (Ser10), which was shown in Figure 2C. Our data collectively show that high-dose finasteride restrains AKT/mTOR signaling, consequentially leading to the suppression of glioblastoma cell proliferation.

**Figure 5:**
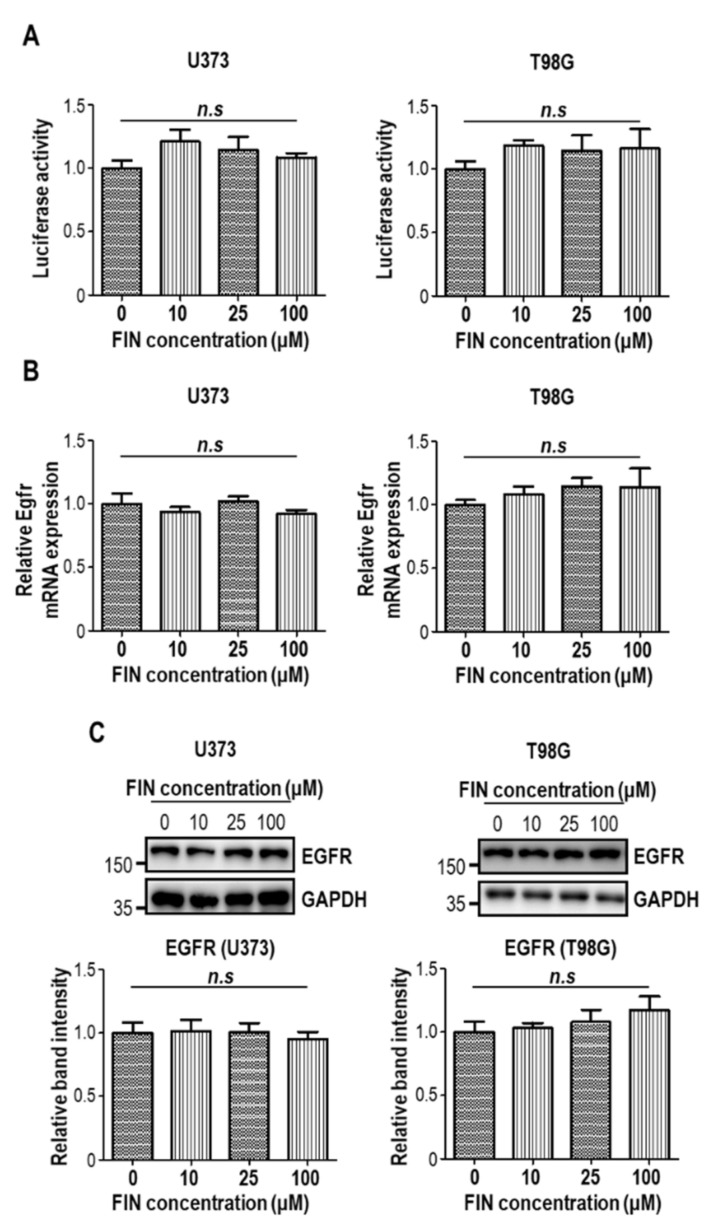
Finasteride does not alter EGFR expression. (**A**) Relative amount of luminance from U373 (left) and T98G (right) cells transfected with *Gaussia* luciferase (GLuc) reporter plasmid-containing EGFR promoter upon treatment of DMSO or finasteride (FIN) at the indicated concentration for 24 h. *n.s.*, not significant. (**B**) The mRNA expression of Egfr in U373 (left) and T98G (right) cells upon vehicle or FIN treatment, as measured by quantitative RT-PCR analysis. The mRNA level of vehicle-treated cells was set to 1. *n.s.*, not significant. (**C**) Immunoblot analysis of EGFR in U373 (left) or T98G (right) cells after treatment with vehicle or FIN at the indicated concentration for 24 h. GAPDH was used as a loading control. The relative band intensities of the EGFR are shown below. The intensities of vehicle-treated samples were arbitrarily set to 1.

Epidermal growth factor receptor (EGFR) has been regarded as a potent oncogene and clinical marker in glioblastoma [28]. Diverse *EGFR* alterations, including point mutation, intragenic deletion, amplification, and overexpression, were documented in the literature [1]. Given that excessive EGFR level enhances the proliferative capacity of glioblastoma [29], we additionally tested whether finasteride could modulate EGFR expression. When we measured EGFR promoter-driven luciferase activities with or without finasteride treatment, we could not detect any difference in EGFR promoter activity upon finasteride administration in both U373 and T98G cells (Figure 5A). Consistent with this data, finasteride did not change EGFR mRNA (Figure 5B) and protein levels (Figure 5C).

### 3.5. Finasteride Seems Less Effective in Inducing DNA Damage than Temozolomide

**Figure 6:**
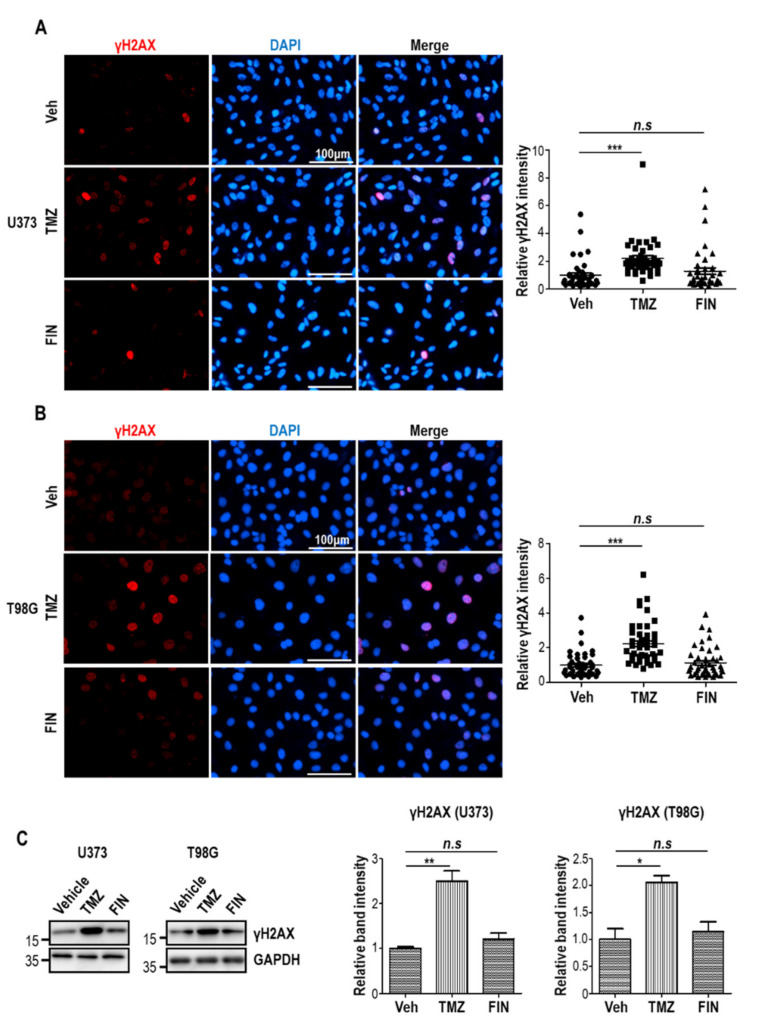
High-dose finasteride shows negligible effect on DNA damage in glioblastoma cells. (A and B) U373 (**A**) and T98G (**B**) cells were treated with DMSO or temozolomide (TMZ) or 100 μM finasteride (FIN) for 24 h, and they were immunostained with a γH2AX antibody (red). Nuclear DAPI staining is presented in blue (left). The relative signal intensities of γH2AX are shown (right). Each dot means an individual cell. (**C**) Immunoblot analysis of γH2AX in U373 (left) or T98G (right) cells upon treatment of DMSO or TMZ or 100 μM FIN for 24 h. GAPDH was used as a loading control. The relative band intensities of γH2AX are represented. The intensities of vehicle-treated samples were arbitrarily set to 1. * *p* < 0.05, ** *p* < 0.01, *** *p* < 0.001.

Temozolomide shows cytotoxicity against glioblastoma by inducing DNA damage. To test whether finasteride could also facilitate the DNA damage response, we performed immunofluorescence staining by utilizing an antibody against the γH2AX, a sensitive marker of DNA detriment, in U373 and T98G cells after treatment with either vehicle or temozolomide or finasteride for 24 h. As expected, temozolomide significantly upregulated the relative γH2AX intensities. However, finasteride failed to increase γH2AX level, revealing that finasteride did not effectively cause DNA damage response (Figure 6A,B). These data were additionally confirmed by immunoblotting. Clearly, compared to temozolomide that dramatically increased γH2AX level, finasteride did not significantly induce H2AX phosphorylation (Figure 6C). Our data collectively show that finasteride demonstrates anti-tumor activity against glioblastoma by suppressing proliferative potential, rather than inducing DNA damage.

## 4. Discussion

Drug discovery and development is time/money-consuming and a risky process. Moreover, finding new drugs to treat abnormalities in the CNS is particularly difficult due to extra obstacle such as the blood–brain barrier. Therefore, drug repurposing (or repositioning) is attractive and competitive strategy to fulfill unmet clinical needs in terms of both time and cost. In this study, we tested a possibility of using finasteride to treat glioblastoma. Finasteride is an FDA-approved medication for the treatment of benign prostate hyperplasia and androgenic alopecia. However, in recent years, a few studies have suggested that finasteride shows some potential as a new glioblastoma drug [30,31]. However, previous studies failed to demonstrate the detailed intracellular mechanisms of finasteride. Furthermore, compared to temozolomide, the pros and cons of finasteride have not been determined so far.

Here, we tested the antitumor activity of finasteride on glioblastoma. Finasteride reduced the stemness of cancer stem-like cells and suppressed the proliferating capacity of glioblastoma. Mechanistically, finasteride downregulated intracellular ROS level by increasing expression of antioxidant genes, presumably leading to inefficient β-catenin accumulation. Lowered β-catenin resulted in the diminution of sustainability and cell growth in glioblastoma. In this study, we deliberately utilized two different glioblastoma cell lines, U373 and T98G, which have disparate characteristics, to check the generalized activity of finasteride. In the promoter region of hTERT, C228T mutation is present in U373 but not in T98G cell. Instead, C250T mutation is present in T98G but absent in U373 cell. For this reason, hTERT mRNA expression level is much higher in T98G than U373 [32]. While U373 cells have a methylated MGMT promoter, T98G cells have wildtype unmethylated MGMT promoter. Therefore, MGMT proteins exist abundantly in T98G, compared to U373. As expected, it was reported that the percentage of γH2AX-positive cells after irradiation decreased rapidly in T98G cells. In contrast, the proportion of γH2AX-positive cells after irradiation sustained until 24 h in U373 [33]. For this reason, while U373 cell was regarded as temozolomide-sensitive cell, T98G cell was known to be relatively resistant to temozolomide [34]. Furthermore, from a metabolic point of view, U373 recapitulates the mitochondrial metabolism-related parameters of primary glioblastoma, but T98G replicates the glycolysis metabolism of primary glioblastoma [35]. Most importantly, finasteride commonly showed antitumor and antioxidant activity in both U373 and T98G cells.

In tumor physiology, ROS can orchestrate both pro-tumorigenic and antitumor pathways in a context-dependent manner. It was previously reported that excessive ROS production by GSH depletion led to the cytotoxicity on colorectal cancer and glioblastoma [36,37]. In contrast, targeted inhibition of xCT, a critical regulator in GSH synthesis, could reduce antioxidant capacity as well as tumor growth and invasiveness in glioma [38]. For these reasons, tumor cells seem to maintain suitable “tumor-promoting ROS levels” to increase their proliferation and survival capacity. This complexity regarding ROS regulation would be one of major challenges for the development of novel therapeutic strategies against diverse types of cancers. In the present study, we suggest that finasteride might cause an imbalance in ROS level to suppress the propagating potential of glioblastoma cells.

Nevertheless, our study has some limitations. First, the antioxidant and antiproliferative function of finasteride was clearly observed at a high dosage. We tried 100 μM finasteride as a maximal concentration, which corresponds to 1000 times the peak plasma levels when men take finasteride at the recommended dose. Although finasteride has been regarded as generally safe and shows a relatively low side effect profile, the concentration used in our in vitro study is hardly achievable in humans. Therefore, following studies should devise a local delivery method (by using biocompatible and biodegradable materials) for minimizing systemic toxicity. Second, because our results showed that finasteride lowered β-catenin level and reduced the stemness of cancer-stem like cells, it would be possible that finasteride might show the anti-proliferative activity against normal adult stem cells. Third, given that finasteride did not effectively induce DNA damage or kill tumor cells in glioblastoma, finasteride could not be used alone. However, we tested a possibility that finasteride has the potential to be developed as an anti-glioblastoma drug through further in vivo experiments and clinical trials.

## Figures and Tables

**Table 1:** Primer sets used in quantitative RT-PCR.

Gene Name	Sequence (5’ to 3’)
*hRPL32*	GAAGTTCCTGGTCCACAACG GCGATCTCGGCACAGTAAG
*hSox2*	GAGCTTTGCAGGAAGTTTGC GCAAGAAGCCTCTCCTTGAA
*hEGFR*	TCCCCGTAATTATGTGGTGAC AGGCCCTTCGCACTTCTTAC

## Data Availability

Not applicable.

## Supplementary Materials

The following are available online at https://www.mdpi.com/article/10.3390/pharmaceutics13091410/s1, Figure S1: Transcriptome analysis of finasteride-treated glioblastoma cells, and Figure S2: The effect of finasteride on cell cycle progression.
