# Supplementary material for: Antioxidant and Antiproliferative Activity of Finasteride against Glioblastoma Cells
Source: Pharmaceutics. 2021 Sep 6;13(9):1410. doi: 10.3390/pharmaceutics13091410 (PMC8469955; doi:10.3390/pharmaceutics13091410)
Supplement: Supplementary file 1 [file pharmaceutics-13-01410-s001.zip › pharmaceutics-1301511-supplementary.pdf]

Supplementary Figure S1

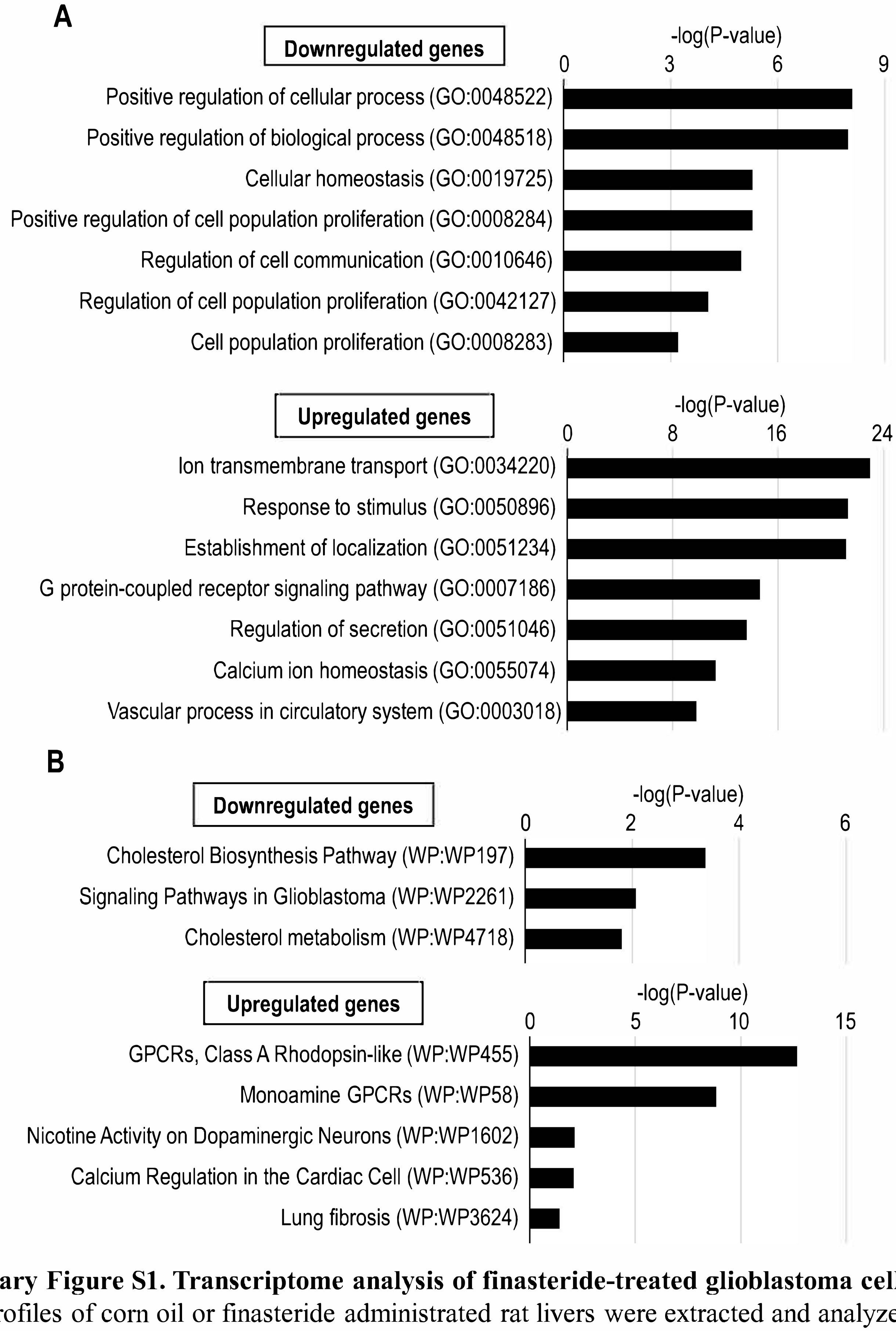

Supplementary Figure S1. Transcriptome analysis of finasteride-treated glioblastoma cells. (A and B) mRNA profiles of corn oil or finasteride administrated rat livers were extracted and analyzed from NCBI GEO profile database (GEO accession: GSE8251). Determination of upregulated genes was based on fold-change of the expression level > 2 and a *p*-value < 0.05. Determination of downregulated genes was based on fold-change of the expression level < 0.5 and a *p*-value < 0.05. GO enrichment analysis for the biological process (A) and biological pathway analysis (B) of up- or down-regulated genes post finasteride treatment were conducted by utilizing g:Profiler.

Supplementary Figure S2

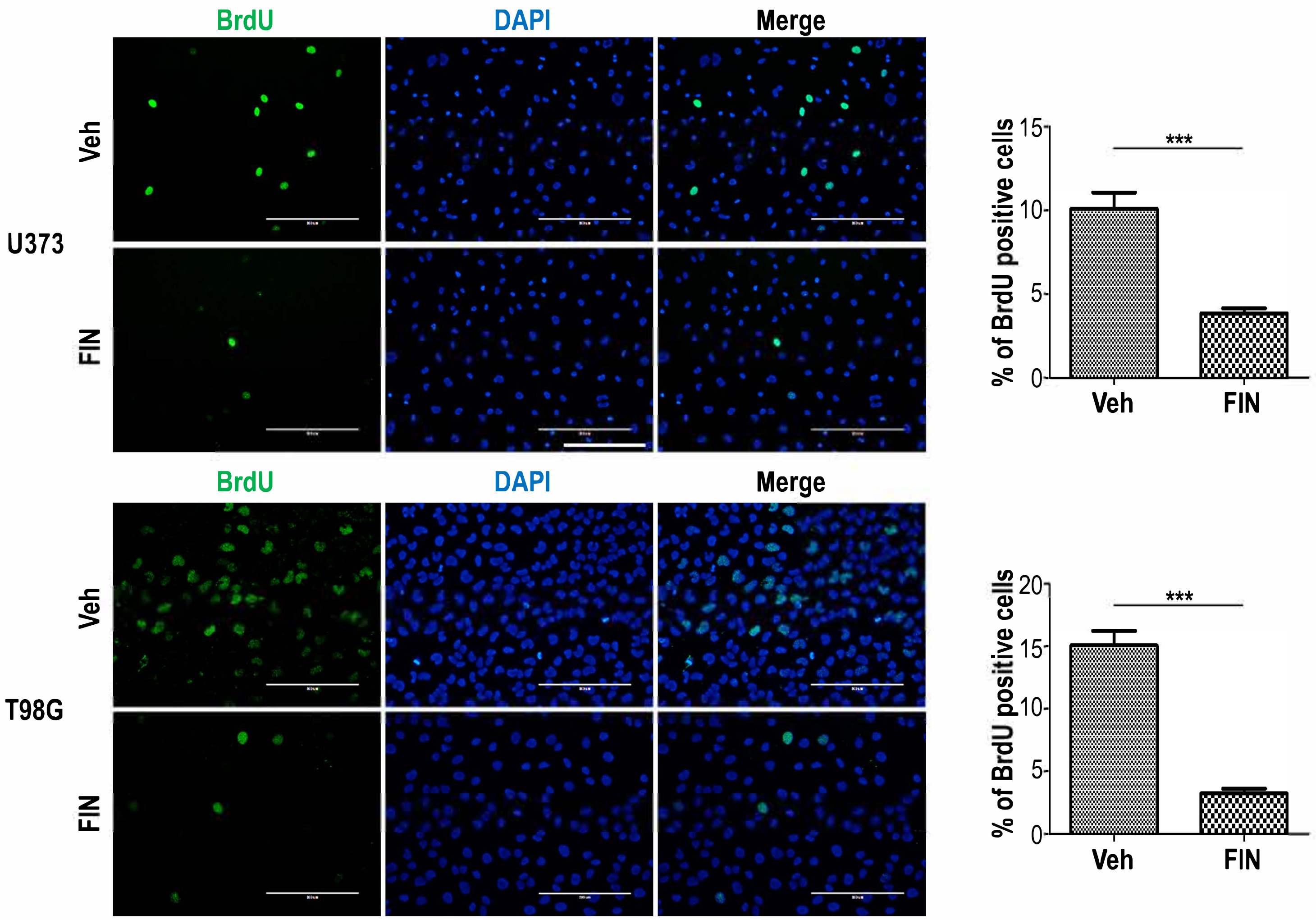

Supplementary Figure S2. The effect of finasteride on cell cycle progression. BrdU staining was performed in vehicle- or FIN-treated cells. Green signals indicated BrdU-positive cells. Nuclear DAPI (4',6-diamidino-2-phenylindole) staining is shown in blue (left). The quantification of BrdU-positive cell proportions in vehicle- or FIN-treated cells (right).
